# Supplementary material for: Projection of dengue fever transmissibility under climate change in South and Southeast Asian countries
Source: PLoS Negl Trop Dis. 2024 Apr 29;18(4):e0012158. doi: 10.1371/journal.pntd.0012158 (PMC11081495; doi:10.1371/journal.pntd.0012158)
Supplement: S1 Text — (DOCX) [file pntd.0012158.s001.docx]

**S1 Text.** Supplementary methodology

Weekly temperature and rainfall projections

Location-specific projected weekly mean temperature (*T_ij_*) and total rainfall (*R_ij_*) were calculated by:

$$T_{ij}=T_{i}+(T_{proj\_j}-T_{avg\_j})$$

$$R_{ij}=R_{i}*(R_{proj\_j}/R_{sum\_j})$$

To establish a baseline, the study calculated the nine-year averaged weekly mean temperature and total rainfall observations in each location. To obtain the projected weekly mean temperature (*T_ij_*) series for the *i*-th week in the *j*-th month in a location, the monthly mean temperature difference between the projection (*T_proj_j_*) and baseline (*T_avg_j_*) temperature was added to the observed baseline weekly temperature (*T_i_)* in the same month *j*. The ratio between projected (*R_proj_j_*) and baseline (*R_sum_j_*) total rainfall in month *j* was multiplied by observed baseline weekly total rainfall (*R_i_*) in the same month to get the projected weekly rainfall (*R_ij_*) series.
