# Supplementary material for: Projection of dengue fever transmissibility under climate change in South and Southeast Asian countries
Source: PLoS Negl Trop Dis. 2024 Apr 29;18(4):e0012158. doi: 10.1371/journal.pntd.0012158 (PMC11081495; doi:10.1371/journal.pntd.0012158)
Supplement: S1 Table — (DOCX) [file pntd.0012158.s002.docx]

**S1 Table.** Data sources

| **Weekly Dengue Cases** | |
| --- | --- |
| Singapore | <https://data.gov.sg/dataset/weekly-number-of-dengue-and-dengue-haemorrhagic-fever-cases?resource_id=c6db5eb2-6daf-4d3b-b571-7bc16c8f03c4> |
| Sri Lanka | <http://www.epid.gov.lk/web/index.php?option=com_content&view=article&id=148&Itemid=449&lang=en> |
| Malaysia | <https://www.moh.gov.my/index.php/database_stores/store_view/17?items=25&page=1> |
| Thailand | <http://doe.moph.go.th/surdata/index.php> |
| **Daily Meteorological Data** | |
| NCEI | <https://www.ncei.noaa.gov/metadata/geoportal/rest/metadata/item/gov.noaa.ncdc%3AC00516/html> |
