# Supplementary material for: Projection of dengue fever transmissibility under climate change in South and Southeast Asian countries
Source: PLoS Negl Trop Dis. 2024 Apr 29;18(4):e0012158. doi: 10.1371/journal.pntd.0012158 (PMC11081495; doi:10.1371/journal.pntd.0012158)
Supplement: S2 Table — (DOCX) [file pntd.0012158.s003.docx]

**S2 Table.** List of 11 included GCMs

| **ID** | **Model name** | **Model Center** |
| --- | --- | --- |
| 1 | ACCESS-CM2 | Commonwealth Scientific and Industrial Research Qrganization, Australia |
| 2 | ACCESS-ESM1-5 |  |
| 3 | CanESM5 | Canadian Center for Climate Modelling and Analysis, Canada |
| 4 | EC-Earth3-Veg | EC-EARTH consortium, Europe-wide consortium |
| 5 | INM-CM4-8 | Institute for Numerical Mathematics, Russian Academy of Science, Russia |
| 6 | INM-CM5-0 |  |
| 7 | IPSL-CM6A-LR | Institut Pierre-Simon Laplace, France |
| 8 | MICRO6 | Japan Agency for Marine-Earth Science and Technology, Atmosphere and Ocean Research Institute, The University of Tokyo, National Institute for the Environmental Studies, and RIKEN Center for Computational Science, Japan |
| 9 | MPI-ESM1-2-HR | Max Planck Institute for Meteorology, Germany |
| 10 | MPI-ESM1-2-LR |  |
| 11 | MRI-ESM2-0 | Meteorological Rresearch Institute, Japan |
