# Supplementary material for: Projection of dengue fever transmissibility under climate change in South and Southeast Asian countries
Source: PLoS Negl Trop Dis. 2024 Apr 29;18(4):e0012158. doi: 10.1371/journal.pntd.0012158 (PMC11081495; doi:10.1371/journal.pntd.0012158)
Supplement: S3 Table — (DOCX) [file pntd.0012158.s004.docx]

**S3 Table.** Basic characteristics and model performance in 30 study locations

| **Region** | **Cases** | **Incidence (/10,000)** | **Temperature (°C)** | **Weekly rainfall (mm)** | **Fitting** | | **Forecasting** | |
| --- | --- | --- | --- | --- | --- | --- | --- | --- |
|  |  |  |  |  | **Correlation** | **RMSE** | **Correlation** | **RMSE** |
| Singapore | 126962 | 25.34 | 27.87 (24.92-29.94) | 43.36 (0-175.71) | 0.72 | 0.21 | 0.38 | 0.33 |
| Sri Lanka |  |  |  |  |  |  |  |  |
| Jaffna | 27523 | 50.22 | 28.97 (24.44-33.89) | 25.09 (0-442.20) | 0.55 | 0.61 | 0.53 | 0.61 |
| Trincomalee | 12249 | 32.67 | 28.61 (24.61-32.49) | 29.99 (0-334.80) | 0.58 | 0.90 | 0.47 | 0.73 |
| Anuradhapura | 6625 | 8.05 | 27.96 (23.80-32.00) | 29.08 (0-445.52) | 0.42 | 0.91 | 0.12 | 0.71 |
| Puttalam | 15727 | 21.56 | 27.81 (23.91-30.86) | 24.41 (0-348.49) | 0.59 | 0.91 | 0.21 | 0.86 |
| Batticaloa | 19754 | 38.92 | 28.53 (25.17-31.49) | 32.94 (0-647.45) | 0.62 | 0.76 | 0.25 | 0.75 |
| Kurunegala | 26068 | 17.22 | 28.44 (23.95-32.55) | 39.04 (0-505.40) | 0.53 | 0.63 | 0.38 | 0.62 |
| Hambantota | 8851 | 15.25 | 27.96 (24.46-30.84) | 19.90 (0-258.06) | 0.46 | 0.69 | 0.10 | 0.67 |
| Kandy | 39117 | 29.94 | 28.06 (24.61-30.50) | 48.22 (0-372.49) | 0.53 | 0.92 | 0.28 | 0.63 |
| Ratnapura | 22548 | 21.88 | 26.58 (24.19-29.03) | 69.00 (0-545.59) | 0.64 | 0.86 | 0.28 | 0.85 |
| Colombo | 112757 | 52.11 | 28.06 (24.61-30.50) | 48.22 (0-372.49) | 0.69 | 0.42 | 0.31 | 0.69 |
| Badulla | 9080 | 11.71 | 23.10 (19.26-27.69) | 35.21 (0-380.49) | 0.50 | 1.00 | 0.27 | 0.74 |
| Galle | 18482 | 18.44 | 27.66 (25.30-30.64) | 44.04 (0-304.80) | 0.40 | 0.92 | 0.11 | 1.01 |
| Thailand |  |  |  |  |  |  |  |  |
| Chiang Mai | 35216 | 22.90 | 26.91 (17.60-34.40) | 20.19 (0-216.00) | 0.78 | 0.56 | 0.68 | 0.61 |
| Chiang Rai | 30174 | 26.99 | 25.32 (14.70-31.92) | 32.40 (0-218.69) | 0.86 | 0.63 | 0.70 | 0.86 |
| Mae Hong Son | 7590 | 31.75 | 26.70 (18.42-33.89) | 22.72 (0-131.45) | 0.75 | 0.67 | 0.48 | 0.96 |
| Phichit | 5557 | 11.35 | 28.34 (19.69-34.54) | 21.18 (0-206.50) | 0.56 | 0.70 | 0.49 | 0.72 |
| Nakhon Sawan | 13457 | 14.01 | 28.62 (20.53-34.43) | 23.43 (0-172.59) | 0.75 | 0.55 | 0.48 | 0.7 |
| Roi Et | 16668 | 14.17 | 27.40 (18.59-34.52) | 27.88 (0-491.80) | 0.76 | 0.61 | 0.53 | 0.77 |
| Loei | 8885 | 15.51 | 26.48 (16.54-33.25) | 23.62 (0-304.32) | 0.73 | 0.69 | 0.43 | 0.83 |
| Nakhon Phanom | 5769 | 8.99 | 28.49 (19.52-36.13) | 43.48 (0-416.40) | 0.72 | 0.78 | 0.25 | 0.87 |
| Malaysia |  |  |  |  |  |  |  |  |
| Kelantan | 40421 | 25.16 | 27.47 (24.67-30.40) | 50.90 (0-667.26) | 0.78 | 0.40 | 0.54 | 0.51 |
| WPKL&Putrajaya | 70439 | 39.85 | 27.89 (25.69-29.81) | 40.30 (0-240.28) | 0.45 | 0.30 | 0.26 | 0.26 |
| Melaka | 16544 | 20.46 | 27.78 (25.20-30.15) | 37.38 (0-243.84) | 0.46 | 0.36 | 0.18 | 0.47 |
| Pahang | 18362 | 12.49 | 27.20 (24.15-29.64) | 52.18 (0-838.71) | 0.54 | 0.31 | 0.23 | 0.37 |
| Perak | 39054 | 17.53 | 27.66 (25.39-30.20) | 35.50 (0-399.03) | 0.60 | 0.26 | 0.23 | 0.31 |
| P.Pinang | 27324 | 17.60 | 28.10 (25.84-30.01) | 36.71 (0-312.42) | 0.54 | 0.30 | 0.34 | 0.47 |
| Sabah | 24927 | 7.25 | 27.56 (24.76-29.25) | 49.36 (0-198.46) | 0.66 | 0.27 | 0.37 | 0.28 |
| Sarawak | 16040 | 6.56 | 27.03 (24.32-29.05) | 68.16 (0-249.36) | 0.60 | 0.30 | 0.13 | 0.47 |
| Selangor | 409704 | 72.35 | 28.17 (25.60-30.67) | 56.73 (0-246.38) | 0.67 | 0.16 | 0.73 | 0.14 |

* Temperature and rainfall are shown as mean value and range. WPKL&Putrajaya: Wilayah Persekutuan Kuala Lumpur & Putrajaya. P.Pinang: Pulau Pinang.
