# Supplementary material for: Projection of dengue fever transmissibility under climate change in South and Southeast Asian countries
Source: PLoS Negl Trop Dis. 2024 Apr 29;18(4):e0012158. doi: 10.1371/journal.pntd.0012158 (PMC11081495; doi:10.1371/journal.pntd.0012158)
Supplement: S4 Table — (DOCX) [file pntd.0012158.s005.docx]

**S4 Table.** DF epidemiology, temperature, and rainfall conditions in four countries

|  | **2012** | **2013** | **2014** | **2015** | **2016** | **2017** | **2018** | **2019** | **2020** | **Total** |
| --- | --- | --- | --- | --- | --- | --- | --- | --- | --- | --- |
| Singapore |  |  |  |  |  |  |  |  |  |  |
| DF cases | 4,766 | 22,497 | 17,889 | 11,304 | 13,206 | 2,785 | 3,409 | 16,094 | 35,012 | 12,6962 |
| Incidence rate (/10,000) | 8.97 | 41.66 | 32.70 | 20.41 | 23.55 | 4.96 | 6.04 | 28.21 | 61.58 | 25.34 |
| Mean Temperature (°C) | 27.51 | 27.64 | 27.82 | 28.03 | 28.24 | 27.70 | 27.77 | 28.22 | 27.88 | 27.87 |
| Weekly Rainfall (mm) | 50.12 | 53.35 | 39.51 | 35.17 | 40.41 | 46.41 | 45.15 | 33.63 | 46.37 | 43.36 |
| Sri Lanka (12 locations) |  |  |  |  |  |  |  |  |  |  |
| DF cases | 15,336 | 15,108 | 24,209 | 16,326 | 26,065 | 103,101 | 34,048 | 61,264 | 23,324 | 318,781 |
| Incidence rate (/10,000) | 10.59 | 9.44 | 16.01 | 11.68 | 17.00 | 76.62 | 28.57 | 46.45 | 22.12 | 26.50 |
| Mean Temperature (°C) | 27.51 | 27.34 | 27.43 | 27.65 | 28.22 | 27.63 | 27.40 | 27.70 | 27.91 | 27.64 |
| Weekly Rainfall (mm) | 35.49 | 34.29 | 43.38 | 43.00 | 29.83 | 36.77 | 36.66 | 42.33 | 32.27 | 37.10 |
| Thailand (8 locations) |  |  |  |  |  |  |  |  |  |  |
| DF cases | 9,039 | 38,523 | 3,527 | 14,434 | 10,346 | 6,764 | 11,304 | 18,213 | 11,166 | 123,316 |
| Incidence rate (/10,000) | 11.36 | 49.40 | 6.78 | 18.57 | 14.43 | 9.08 | 15.46 | 21.31 | 17.47 | 18.21 |
| Mean Temperature (°C) | 27.22 | 27.07 | 27.16 | 27.44 | 27.26 | 27.01 | 26.95 | 27.77 | 27.68 | 27.28 |
| Weekly Rainfall (mm) | 26.80 | 28.49 | 26.44 | 24.33 | 29.56 | 33.48 | 28.64 | 23.00 | 20.96 | 26.86 |
| Malaysia (9 locations) |  |  |  |  |  |  |  |  |  |  |
| DF cases | 18,348 | 35,670 | 93,269 | 100,109 | 86,501 | 70,875 | 69,666 | 114,420 | 73,957 | 662,815 |
| Incidence rate (/10,000) | 7.11 | 12.02 | 34.72 | 32.75 | 28.93 | 22.22 | 21.07 | 35.65 | 24.77 | 24.36 |
| Mean Temperature (°C) | 27.36 | 27.53 | 27.56 | 27.69 | 28.02 | 27.51 | 27.52 | 27.88 | 27.78 | 27.65 |
| Weekly Rainfall(mm) | 49.32 | 45.13 | 46.68 | 42.84 | 45.52 | 53.28 | 52.44 | 42.46 | 49.54 | 47.47 |

* The country-level DF incidence rate shows annual average of the location-specific incidence rates, which were calculated as dividing the total number of cases in a year by the mid-year population in each location. The maximum temperature and weekly rainfall were annual averages of the location-specific weekly mean temperature and weekly cumulative rainfall in each country.
