# Supplementary material for: Projection of dengue fever transmissibility under climate change in South and Southeast Asian countries
Source: PLoS Negl Trop Dis. 2024 Apr 29;18(4):e0012158. doi: 10.1371/journal.pntd.0012158 (PMC11081495; doi:10.1371/journal.pntd.0012158)
Supplement: S5 Table — (DOCX) [file pntd.0012158.s006.docx]

**S5 Table.** Projected DF epidemic duration in 2030s under climate change

| **Region** | **SSP 126** | **SSP245** | **SSP 585** |
| --- | --- | --- | --- |
| Singapore | 31 | 31 | 32 |
| Sri Lanka |  |  |  |
| Jaffna | 40 | 40 | 40 |
| Trincomalee | 20 | 19 | 20 |
| Anuradhapura | 25 | 25 | 25 |
| Puttalam | 37 | 35 | 35 |
| Batticaloa | 30 | 31 | 30 |
| Kurunegala | 32 | 32 | 32 |
| Hambantota | 37 | 37 | 37 |
| Kandy | 18 | 18 | 19 |
| Ratnapura | 28 | 28 | 28 |
| Colombo | 34 | 35 | 35 |
| Badulla | 25 | 25 | 25 |
| Galle | 35 | 35 | 35 |
| Thailand |  |  |  |
| Chiang Mai | 18 | 18 | 18 |
| Chiang Rai | 26 | 26 | 25 |
| Mae Hong Son | 27 | 26 | 28 |
| Phichit | 23 | 23 | 23 |
| Nakhon Sawan | 28 | 28 | 27 |
| Roi Et | 35 | 35 | 35 |
| Loei | 21 | 21 | 21 |
| Nakhon Phanom | 24 | 24 | 24 |
| Malaysia |  |  |  |
| Kelantan | 33 | 32 | 31 |
| WPKL&Putrajaya | 30 | 29 | 29 |
| Melaka | 38 | 37 | 35 |
| Pahang | 34 | 35 | 35 |
| Perak | 45 | 45 | 45 |
| P.Pinang | 40 | 38 | 36 |
| Sabah | 45 | 45 | 45 |
| Sarawak | 33 | 33 | 31 |
| Selangor | 37 | 37 | 37 |

* DF epidemic duration was defined as number of weeks with weekly R_t_ higher than unity in a year. WPKL&Putrajaya: Wilayah Persekutuan Kuala Lumpur & Putrajaya. P.Pinang: Pulau Pinang.
