# Supplementary figures and images for: Projection of dengue fever transmissibility under climate change in South and Southeast Asian countries
Source: PLoS Negl Trop Dis. 2024 Apr 29;18(4):e0012158. doi: 10.1371/journal.pntd.0012158 (PMC11081495; doi:10.1371/journal.pntd.0012158)

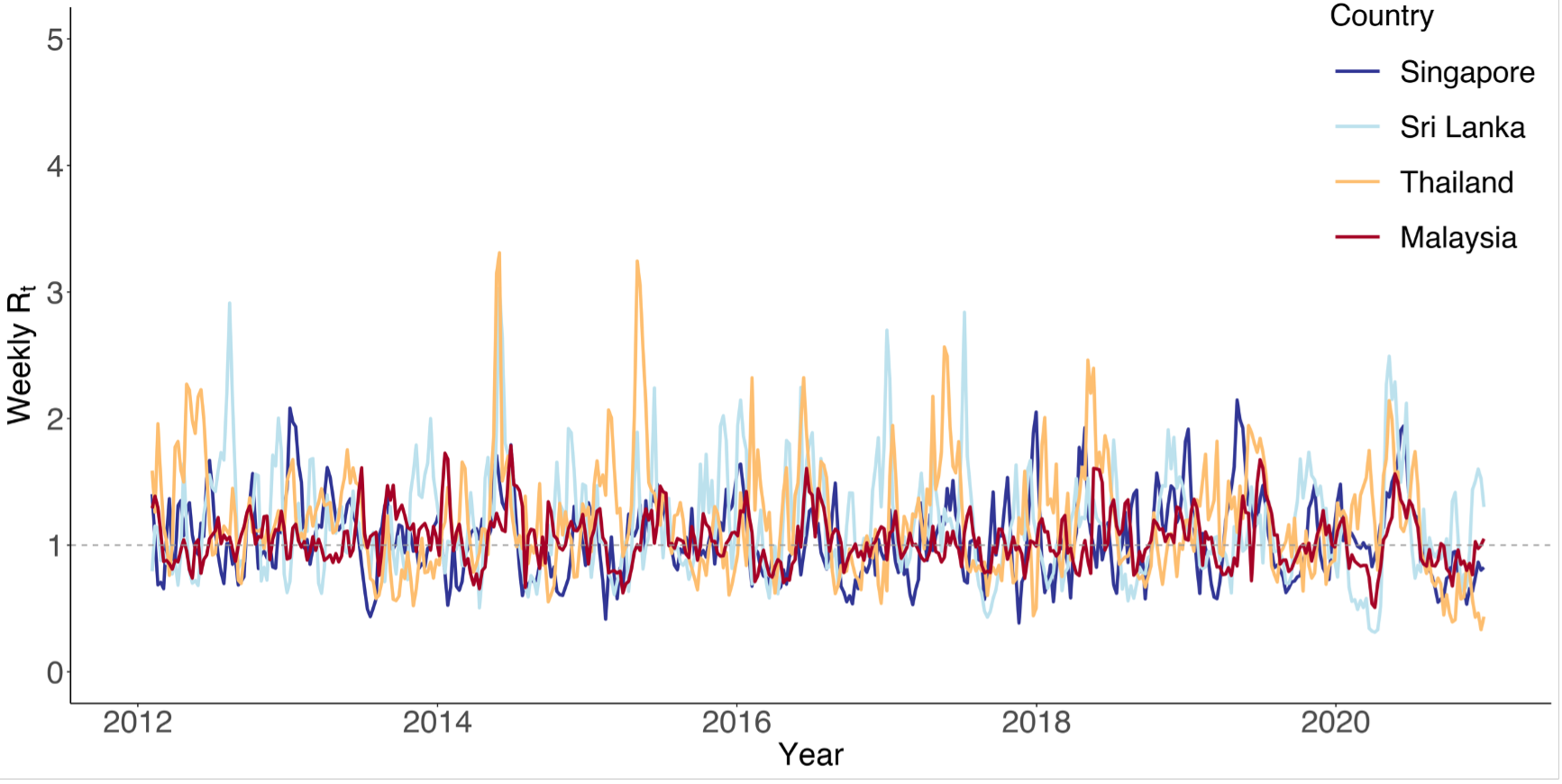

Supplement: S1 Fig — (TIFF) [file pntd.0012158.s007.tiff]

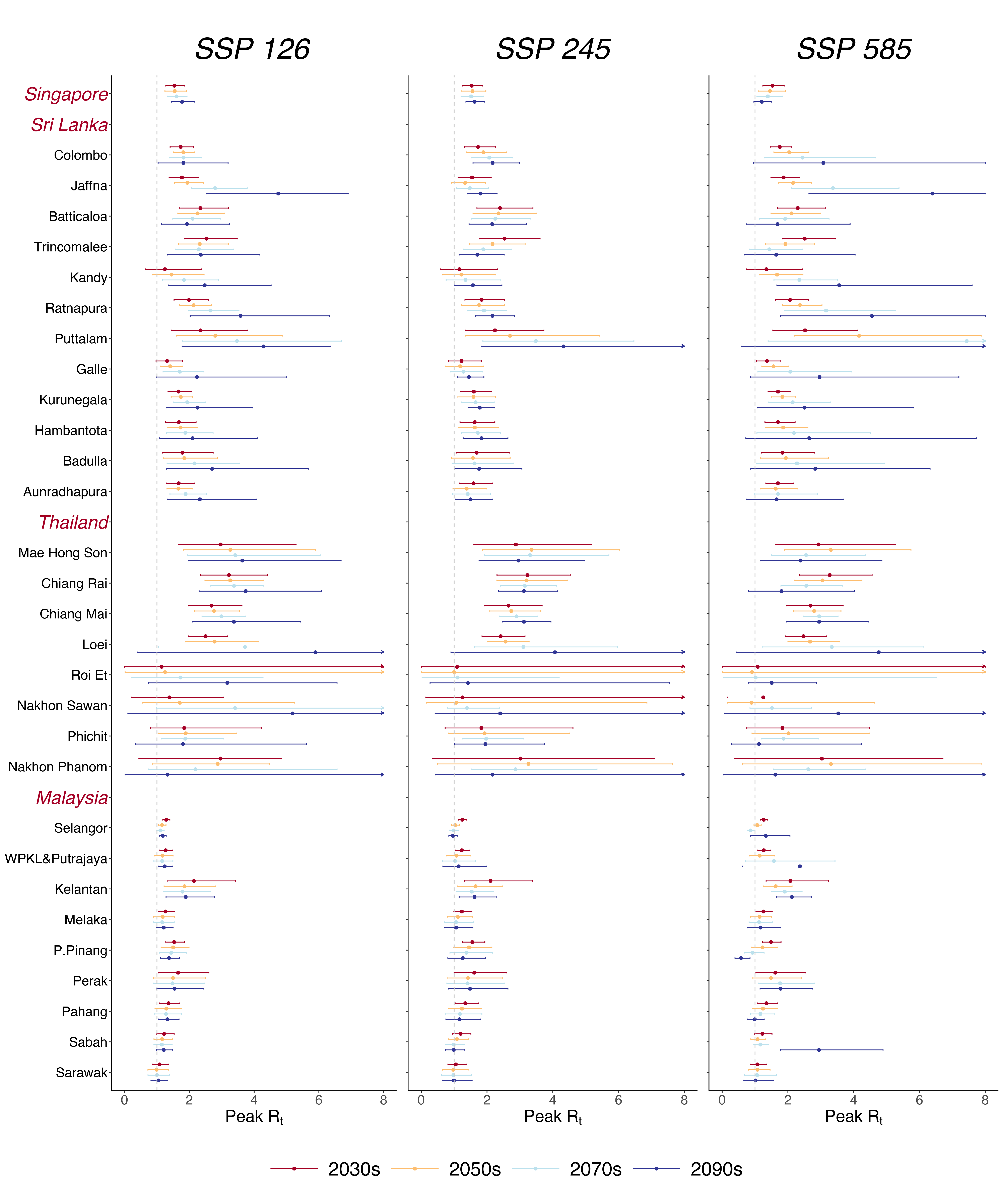

Supplement: S2 Fig — WPKL&Putrajaya: Wilayah Persekutuan Kuala Lumpur & Putrajaya. P.Pinang: Pulau Pinang. Estimated by models accounting for the population. (TIFF) [file pntd.0012158.s008.tiff]

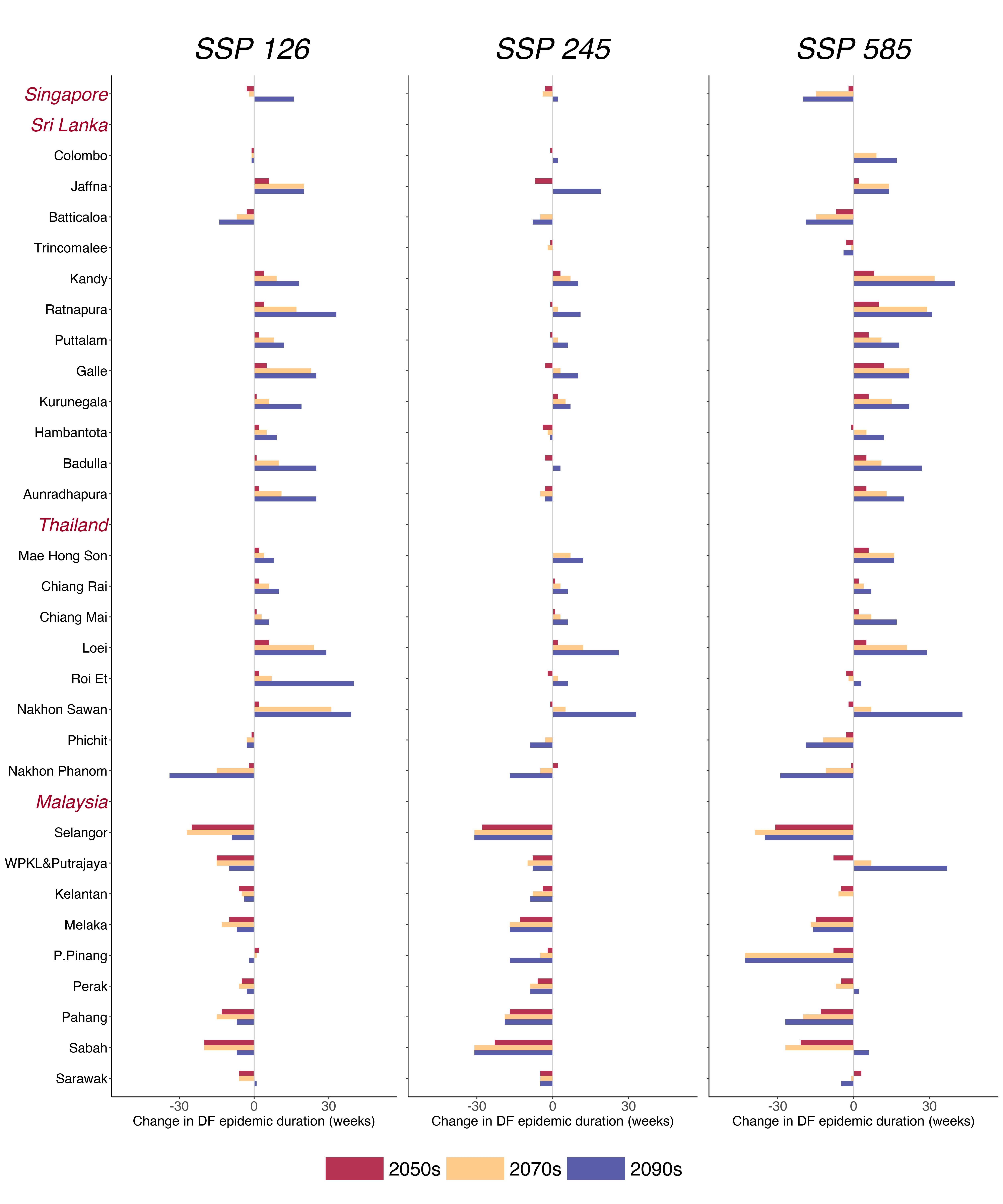

Supplement: S3 Fig — DF epidemic duration was defined as number of weeks with Rt higher than unity in a year. WPKL&Putrajaya: Wilayah Persekutuan Kuala Lumpur & Putrajaya. P.Pinang: Pulau Pinang. Estimated by models accounting for the population. (TIFF) [file pntd.0012158.s009.tiff]
