# Supplementary material for: Projection of dengue fever transmissibility under climate change in South and Southeast Asian countries
Source: PLoS Negl Trop Dis. 2024 Apr 29;18(4):e0012158. doi: 10.1371/journal.pntd.0012158 (PMC11081495; doi:10.1371/journal.pntd.0012158)
Supplement: Attachment — Submitted filename: PLoS_NTDS_10jan24.docx [file pntd.0012158.s010.docx]

The manuscript “Projection of dengue fever transmissibility under climate change in South and Southeast Asian countries” evaluates projections of dengue in four countries of South and Southeast Asia. The approach focuses the development of climate-based prediction models of dengue and applies the best models (based on RMSE) to generate dengue Rt and epidemic duration projections for each country under three climate scenarios. These scenarios are based on the low, middle and high levels of greenhouse gas emissions. Authors found substantial variability in projections of Rt and epidemic duration across geographies and the strongest decline in Rt occurring in mid-high greenhouse gas emission scenarios. Findings highlight how changes in climate and the environment can have unexpected impacts on dengue in areas with substantial dengue transmission, such as in the South and Southeastern Asian region. Overall, the manuscript was clear and well-written. The findings of the study are very interesting, but the Methods and Results needs additional clarification or expansion, and particularly the within-country comparisons. The topic is of importance, particularly in evaluation how dengue many change with respect to intensity and duration. Comments are described below:

**Major comments:**

1. Page 6, line 95-98: Some additional context to these climate scenarios would be helpful to orient the reader. It’s not immediately clear what the high emission scenario is prior to getting to it in the Methods section. Are these scenarios part of IPCC or oriented to potential region/country-specific reductions/targets of green-house gasses? A little expansion into the general relationship between greenhouse gasses and temperature/rainfall might help to clarify.
2. Page 6, line 108: Referencing some of the limited studies would help show what has been done prior to this study.
3. Page 8 Methods: line 139: the current description of how the data for the climate periods needs clarification– from the description, it seems like for each period, there is only 1 average value for a location (2020-2041 average), but I would have assumed there was finer resolution of climate data to generate time-series projections for each location for a given period and scenario.
4. Page 10, line 183 – 184: There needs more description as to how the projectionss for the location specific Rt and epidemic durations were generated. Are these projected values reflecting aggregated estimates of multiple time-points for a given location? is it based on 1 aggregate value? From the Rt projections, how were the epidemic duration values obtained? From the description of the climate projections and the description of these projections it is unclear.
5. Page 10, 189: the descriptive table in the supplement is helpful to see some of the within country variability. One other addition to supplement that would help is a map of these countries and where the study locations are (or as a choropleth of the dengue incidence) – I would suspect that there is some geographic clustering of the locations with high dengue incidence, and therefore share some similar climates, but hard to know just by the description.
6. Page 11, line 201 – 206: I’m not convinced that the Rt in Thailand shows distinct seasonality – at the moment, it reads as a country-level observation, but is that based on both the Rt values and peak weeks for all locations within Thailand compared to the other locations? Some comparisons of specific locations might help show these differences a little more. Mention of specific peak weeks (or range of peak weeks) for Sri Lanka and Thailand would also help to compare to Singapore and Malaysia.
7. Figure1 and caption – the caption could use some additional explanatory information. For example, given that the time-series are not showing years, are these average weekly incidences/temperature/rainfall estimates? Are these showing country-aggregated estimates or are these capturing the range of values across the locations within each country?
8. Figure 2 and caption: is the dashed line meant to reference Rt =1? Is there any order to the listing of the locations within each country (i.e. by population, incidence, geographic location)? If these are estimated, are they using the observed data from 2012-2020 or are these predictions?
9. Page 11, line 213-216: First mention of correlation coefficients as part of the model prediction evaluation - Please add these metrics into the Methods. Some additional clarification would help understand the model performance. For example, are these from the testing data, comparing the observed to predicted estimates? Perhaps some mention of which models corresponded to the range of RMSE and correlation values presented would help understand which models performed better.
10. Page 14, lines 266-268: In which ways (both directions and magnitudes) are transmission potential expected to change?
11. Page 15: lines 284: Though the focus is on climate, it would be worth adding in text to discuss non-climate factors that could impact transmission intensity and duration. For example, how much might changes in susceptibility or demographic changes in these projected populations also impact future transmission (e.g., <https://www.pnas.org/doi/10.1073/pnas.2115790119>) or comparisons to other papers that have incorporated non-climate aspects into projections (e.g. https://doi.org/10.1038/s41467-023-43954-0)?
12. Page 15, line 299: A reference for increased severe risk is needed – this recent paper may be useful: <https://www.pnas.org/doi/10.1073/pnas.0709029105>. Some expansion on why there is believed to be a potential shift in risk of severity would help.

**Minor comments:**

1. Page 5, line 90: “shorted extinct incubation period” – perhaps meant extrinsic incubation period?
2. Page 7, line 118-119: are these probable/confirmed cases? Reported symptomatic or hospitalized?
3. Page 8, line 139: Do the periods reported reflect the mid-point of the years of each period? Please clarify.
4. Page 10, line 189-197: Though the averages for incidence/temp/rainfall are reported, providing the IQR values for each country, and perhaps the location with the low and high values would be helpful to better understand how much variability in these there are across the locations.
5. Figure 2: Adding the peak week as a text to the peak week plot could be helpful since its also referenced in the text.
