# Supplementary material for: Projection of dengue fever transmissibility under climate change in South and Southeast Asian countries
Source: PLoS Negl Trop Dis. 2024 Apr 29;18(4):e0012158. doi: 10.1371/journal.pntd.0012158 (PMC11081495; doi:10.1371/journal.pntd.0012158)
Supplement: Attachment — Submitted filename: response to reviewers.docx [file pntd.0012158.s011.docx]

**Response to Reviewers’ Comments**

\## Editor

Q1: The reviewers have made a number of constructive suggestions both on your methods and the explanation of your methods and result, which we encourage you to carefully consider in your revision.

Additionally, we ask you to clarify, both in the manuscript and your response to reviewer comments, how the submitted manuscript is distinct from Wang et al 2023 Impact of climate change on dengue fever epidemics in South and Southeast Asian settings: a modeling study.

A1: Thanks for the construction comments and we have revised our manuscript based on their comments.

We would like to clarify there are many differences between this submission and the previous published article, including:

1) Objective: The primary objective of the published study is to simulate daily dengue fever incidence in four locations under current and future climate using a dynamic compartmental model, and the results help to assess the dengue fever outbreak behaviors (i.e, change of daily incidence) under climate change. However, this submission aims to estimate the dengue fever transmission potential (represented by the reproduction number), which is more related to the transmission dynamics of a disease within a population. Although disease incidence is a crucial measure of disease spread, studying the reproduction number provides insights into the speed and extent of disease transmission, as well as its potential to cause epidemics.
2) Methodology: The published study utilized the well-adopted temperature- and rainfall-dependent parameters and a compartmental model to simulate the dengue infection under different climate change scenarios. Those parameters were determined by summarizing several laboratory and field studies, and they were used to reflect general changes of mosquito behaviors in various environments. Nevertheless, this submission firstly evaluated the location-specific relationship between temperature, rainfall, and dengue fever transmission potential with generalized additive model, one of the classical time-series analysis models, and then we used the estimated associations to project the location-specific changes in the future. Thus, the methodologies of two studies are completely different.
3) Outcome: The published paper used the simulated daily dengue fever incidence as outcome, and higher disease incidence meant more severe disease outbreak. However, current study used the reproduction number of dengue fever as the outcome - If the reproduction number is above 1, it indicates that the disease is spreading, and control measures need to be strengthened. On top of the disease incidence, studying the reproduction number complements this information by providing valuable insights into the transmissibility of the disease and its potential for causing outbreaks.

We also checked for plagiarism between the two studies, and the similarity between this revised manuscript and the Wang et al 2023 is just 4.39% (via VeriGuide).

We have also revised the manuscript and clarified the difference between two studies:

*“To our knowledge, there is limited research on estimating the impact of climate change on dengue fever transmissibility, specifically the reproduction number. This study utilized location specific climate-dengue relationships to predict the disease transmission potential, revealing heterogeneous changes across locations. Wang et al. [39] simulated the change of dengue fever incidence in four South and Southeast Asian locations by using compartmental models with temperature- and rainfall-dependent parameters, and suggested a potential increase in dengue fever peak incidence and longer disease epidemic duration in the future. While disease incidence data is useful for assessing the disease burden and tracking its progression, investigating the reproduction number as the transmissibility outcome complements this information by providing valuable insights into the transmission dynamics and potential of outbreaks.” (Track changes: page 18, line 356-367)*

##Reviewer 1

Q1: Surveillance efforts will vary greatly across the four countries and local sites, and it does not appear the authors have taken this into account in their analyses. Although the sources are listed in the SI, there is no mention of potential reporting bias anywhere in the manuscript. Making cross-country comparisons is therefore problematic.

A1: We acknowledge that the surveillance efforts and reporting practices may vary across the locations, we have attempted to use standardized data sources and methods to minimize the impact of the varied surveillance efforts. For example, we collected the disease data from official weekly surveillance reports, and we have compared and found no apparent difference in the disease diagnose criteria and the infectious disease report procedures across the included locations:

The Ministry of Health (MOH) Singapore modified recommendations for the initial evaluation of the suspected dengue patient. In Singapore, a suspected person will be confirmed with an acute febrile illness when at least two of the following features are shown: headache, eye pain, myalgia, arthralgia, rash, hemorrhagic manifestations, and leukopenia. To confirm an acute infection, the Non-Structural (NS) 1 antigen detection and IgM or IgG detection. The NS-1 antigen is detectable from the first day of the fever onset to up to the 9th day after the fever onset. And the IgM could be detected by the 3^rd^ to 5^th^ day after illness onset.

In Thailand, dengue fever diagnostic criteria were also revised slightly. Firstly, the suspectable people should be the residents or had travelled to a dengue-endemic area before the illness onset. Special symptoms include acute fever accompanied by at least two of the following: headache, myalgia, arthralgia, rash, positive tourniquet test and leucopoenia, with no evidence of plasma leakage. Patients with body temperature equal to or higher than 38 °C, petechiae, ecchymosis, or a positive tourniquet test, thrombocytopenia (platelets < 100,000 cells/mm^3^), and evidence of plasma leakage would be diagnosed as DHF. The most severe DENV infection, dengue shock syndrome (DSS), is confirmed with the same signs and symptoms as DHF, and has circulatory failure.

In Malaysia, dengue cases are diagnosed based on WHO criteria. A probable dengue case was confirmed with an acute febrile illness with at least two clinical manifestations: headache, retro-orbital pain, myalgia, arthralgia, rash, haemorrhagic manifestations, and leukopenia. In addition, supportive serology tests and spatiotemporal correlation with confirmed cases are also considered. A confirmed dengue case is one confirmed by laboratory criteria such as dengue virus isolation, a fourfold rise in antibody titres, virus antigen detection or virus genomic sequence detection.

In Sri Lanka, patients presenting with acute onset of fever and clinical manifestations of headache, especially retro-orbital pain, myalgia /arthralgia, rash (diffuse, erythematous, macular) and haemorrhage. A full blood count is performed to investigate leukopenia, thrombocytopenia and haematocrit (HCT). Fever with at least two of the clinical manifestations and thrombocytopenia are considered sufficient for diagnosing dengue. Patients with evidence of vascular leakage, e.g., pleural effusion and ascites, as ascertained by ultrasound scanning or a rise in the HCT of ≥20%, are classified as having DHF.

In addition to the similar dengue fever diagnose criteria, all four countries required dengue fever cases to be reported within 24 hours. Nevertheless, we acknowledge that this aspect was not explicitly addressed in our analyses, and we have discussed the potential limitations related to surveillance efforts and reporting bias in our revised manuscript. We hope this will help provide a more thorough understanding of the implications of these variations on our analyses.

*“Furthermore, while this study employed standardized data sources and methods to mitigate the effects of differing surveillance efforts, the potential variations in reporting bias among locations were not addressed due to a lack of available data, and this may diminish the precision of the findings.” (Track changes: page 18, line 373-376)*

Q2: The authors state as a limitation that they only consider temperature/rainfall data in their models. Why is this the case? Although socio-economic variables are difficult to obtain projections for at a high spatial resolution, at the very least factors such as population density will have an effect on the varying incidence rates by site and need to be accounted for.

A2: Thanks for the suggestion. Population projections have been incorporated into our analysis to adjust for the potential impact of population size. Specifically, we included the log-transformed observed population data as an offset when estimating the relationship between temperature/rainfall and dengue transmissibility at specific locations. Future temperature and rainfall, as well as population projections of each location, were utilized to predict future dengue fever transmission potential. Compared to the models without population, including population resulted a similar changing trend both in peak transmissibility and the duration of dengue fever outbreak in most of the included locations, and the magnitude of the changes predicted by population-included model would be higher than that estimated without accounting population. We have also revised the manuscript and included the results from models with and without population, and we have indicated the potential limitation of lack of other factors that may affect the disease transmission prediction.

*“Due to lack of enough data, only temperature, rainfall, and population were used to project the DF transmission potential in the future, overlooking other factors that may affect disease transmission dynamics.”(Track changes: page 18, line 368-371)*

Below are the updated model outputs for projected peak *R_t_* under climate change:

Peak reproduction number predicted by models

without population.

Peak reproduction number predicted by models

with population

Disease epidemic duration predicted by models

without population

Disease epidemic duration predicted by models

with population

Q3: The graphs are useful for showing error/uncertainty by country/site, but the paper would greatly benefit from having data displayed in map form (including input variables, incidence rates, and model outputs).

A3: We have mapped the input and output variables, and listed them in the main text. Here is the distribution of the input variables and model outputs.

*“Fig 1. Distribution of DF incidence, mean temperature, and weekly rainfall series in four countries. Panel 1, weekly series box plots. Each box in the plot represents the location-averaged weekly values for the same week of each year across all included locations in the country. The line within the box denotes the median value, while the whiskers on the top and bottom of each box represent the maximum and minimum location-averaged values observed for that week over the span of nine years. Panel 2, spatial distribution of weekly mean temperature (A), weekly total rainfall (B), population density (C), and dengue fever incidence rate (D) in four countries.” (Track changes: page 11, line 215-222)*

*“Fig 3. Projection of the DF peak weekly R_t_ in 30 locations in four countries. Panel 1, projected peak weekly R_t_ and 95% confidence interval of the included locations. Panel 2, special distribution of the projected peak weekly Rt in the future: SSP126-2030s (A), SSP245-2030s (B), SSP585-2030s (C), SSP126-2090s (D), SSP245-2090s (E), and SSP585-2090s (F). WPKL&Putrajaya: Wilayah Persekutuan Kuala Lumpur & Putrajaya. P.Pinang: Pulau Pinang. Estimated by models without accounting for the population.” (Track changes: page 14, line 263-269)*

*“Fig 4. Projected change of DF epidemic duration under climate change. Panel 1, the projected change of epidemic duration in the future compared to the value in 2030s under each SSP scenario (S5 Table). DF epidemic duration was defined as number of weeks with Rt higher than unity in a year. Panel 2, the special distribution of the projected DF epidemic duration in the future: SSP126-2030s (A), SSP245-2030s (B), SSP585-2030s (C), SSP126-2090s (D), SSP245-2090s (E), and SSP585-2090s (F). WPKL&Putrajaya: Wilayah Persekutuan Kuala Lumpur & Putrajaya. P.Pinang: Pulau Pinang. Estimated by models without accounting for the population.” (Track changes: page 15, line 290-297)*

Q4: The limitations are very lightly touched upon. What other variables should be considered besides temperature/rainfall and why have they not been in the current analysis?

A4: We acknowledge that the disease transmission may be affected by several factors other than climate change, such as population density, control interventions, and health care levels. As the projections of varies confounders were not available, we primarily focused only on climate change. However, as population change is predictable, we accounted the change in the population density in our study, and noted that the lack of other variables is a limitation.

*“Due to lack of enough data, only temperature, rainfall, and population were used to project the DF transmission potential in the future, overlooking other factors that may affect disease transmission dynamics. However, the results of this study are still encouraging as we primarily focused on the alteration of DF transmission potential in this century.” (Track changes: page 18, line 368-372)*

\## Reviewer 2

The manuscript “Projection of dengue fever transmissibility under climate change in South and Southeast Asian countries” evaluates projections of dengue in four countries of South and Southeast Asia. The approach focuses the development of climate-based prediction models of dengue and applies the best models (based on RMSE) to generate dengue Rt and epidemic duration projections for each country under three climate scenarios. These scenarios are based on the low, middle and high levels of greenhouse gas emissions. Authors found substantial variability in projections of Rt and epidemic duration across geographies and the strongest decline in Rt occurring in mid-high greenhouse gas emission scenarios. Findings highlight how changes in climate and the environment can have unexpected impacts on dengue in areas with substantial dengue transmission, such as in the South and Southeastern Asian region. Overall, the manuscript was clear and well-written. The findings of the study are very interesting, but the Methods and Results needs additional clarification or expansion, and particularly the within-country comparisons. The topic is of importance, particularly in evaluation how dengue many change with respect to intensity and duration. Comments are described below:

**Major comments:**

Q1: Page 6, line 95-98: Some additional context to these climate scenarios would be helpful to orient the reader. It’s not immediately clear what the high emission scenario is prior to getting to it in the Methods section. Are these scenarios part of IPCC or oriented to potential region/country-specific reductions/targets of green-house gasses? A little expansion into the general relationship between greenhouse gasses and temperature/rainfall might help to clarify.

A1: We have revised the expression and give an explanation to the emission scenarios.

*“Greenhouse gas emission is generally admitted as one of the key contributors to climate change, and the future climate are usually be divided into low, middle, or high emission groups, which represent slow, middle, and rapid temperature, rainfall, and other climate variables changes, respectively.” (Track changes: page 6, line 96-100)*

Q2: Page 6, line 108: Referencing some of the limited studies would help show what has been done prior to this study.

A2: Thanks for the comment. Two previous studies have been referred:

*“Considering the limited studies investigating the disease spread under climate change in South and Southeast Asia [20, 21], this study aimed to project the DF transmission potential in 30 locations across four South and Southeast Asian countries under different climate change scenarios.” (Track changes: page 6, line 112-115)*

*20. Colon-Gonzalez FJ, Gibb R, Khan K, Watts A, Lowe R, Brady OJ: Projecting the future incidence and burden of dengue in Southeast Asia. Nat Commun 2023, 14(1):5439.*

*21.	Colon-Gonzalez FJ, Sewe MO, Tompkins AM, Sjodin H, Casallas A, Rocklov J, Caminade C, Lowe R: Projecting the risk of mosquito-borne diseases in a warmer and more populated world: a multi-model, multi-scenario intercomparison modelling study. Lancet Planet Health 2021, 5(7):e404-e414.*

Q3: Page 8 Methods: line 139: the current description of how the data for the climate periods needs clarification– from the description, it seems like for each period, there is only 1 average value for a location (2020-2041 average), but I would have assumed there was finer resolution of climate data to generate time-series projections for each location for a given period and scenario.

A3: For each period, there is 12 average values (from January to December) for a location under a climate change scenario and a time points. The climate projection used in this study originated from the assessment reports by Intergovernmental Panel on Climate Change (IPCC). Briefly, numerous models that predicted high-resolution gridded climate change based on various assumptions and time scales. This study selected 11 models proven to be accurate for southeast Asia. For each climate change scenario and each time point, the outputs of all selected models were averaged to create a single gridded map containing climate data, and then averaged the gridded climate data within the administrative boundaries to represent the overall climate condition. Climate projections from each model were averaged every 20 years to obtain an overall value depicting the climate condition throughout the specified time period. As this study primarily focused on the general trend in dengue fever transmissibility through this century, utilizing the 2020-2040 averaged projections (referred to as 2030s) is a straightforward method to get a quick overview of the potential trends. Nevertheless, we acknowledge that using model-predicted yearly time-series projections spanning from 2020 to 2040 may lead to more accurate estimations. We have revised the relevant description and identified incorporating fine-scale time-series predictions as a suggestion for future research.

*“Besides, this study used the 20-years averaged climate projections (e.g., 2030s referred to 2020-2040 average) to depict the general climate change condition over that period. However, this approach may overlook variations in climate and other fluctuations throughout the timeframe. It is recommended that future research consider incorporating fine-scale yearly climate projections for more precise and detailed estimations of dengue fever transmission.” (Track changes: page 19, line 380-385)*

Q4: Page 10, line 183 – 184: There needs more description as to how the projections for the location specific Rt and epidemic durations were generated. Are these projected values reflecting aggregated estimates of multiple time-points for a given location? is it based on 1 aggregate value? From the Rt projections, how were the epidemic duration values obtained? From the description of the climate projections and the description of these projections it is unclear.

A4: For each location, we firstly used the observed dengue incidence to calculate the time series of weekly Rt over the nine years, and then evaluated its relationship with weekly temperature and rainfall. Once the model is determined, we can use the weekly temperature and rainfall projection time series to predict the Rt series under different climate patterns. Thus, for each location and each climate change scenario, a weekly Rt series will be predicted, and the epidemic duration was obtained by accounting weeks with weekly Rt >1. We have revised the relevant description.

*“By using the projected series of input variables, the weekly Rt series in the future will be estimated for each location under each climate change scenario and time-period. Two indicators, peak Rt and epidemic duration, were assessed to quantitatively measure the disease transmission potential. Epidemic duration was determined as number of weeks with weekly Rt higher than one in a given year.” (Track changes: page 10, line 190-194)*

Q5: Page 10, 189: the descriptive table in the supplement is helpful to see some of the within country variability. One other addition to supplement that would help is a map of these countries and where the study locations are (or as a choropleth of the dengue incidence) – I would suspect that there is some geographic clustering of the locations with high dengue incidence, and therefore share some similar climates, but hard to know just by the description.

A5: We have mapped the input and output variables, and listed them in the main text. Here is the distribution of the input variables and model outputs.

*“Fig 1. Distribution of DF incidence, mean temperature, and weekly rainfall series in four countries. Panel 1, weekly series box plots. Each box in the plot represents the location-averaged weekly values for the same week of each year across all included locations in the country. The line within the box denotes the median value, while the whiskers on the top and bottom of each box represent the maximum and minimum location-averaged values observed for that week over the span of nine years. Panel 2, spatial distribution of weekly mean temperature (A), weekly total rainfall (B), population density (C), and dengue fever incidence rate (D) in four countries.” (Track changes: page 11, line 215-222)*

Q6: Page 11, line 201 – 206: I’m not convinced that the Rt in Thailand shows distinct seasonality – at the moment, it reads as a country-level observation, but is that based on both the Rt values and peak weeks for all locations within Thailand compared to the other locations? Some comparisons of specific locations might help show these differences a little more. Mention of specific peak weeks (or range of peak weeks) for Sri Lanka and Thailand would also help to compare to Singapore and Malaysia.

A6: We acknowledge that using location-averaged data to reflect the overall changing trend is not reasonable enough. We have revised the description and reported the specific peak weeks and the corresponding values to help to facilitate comparisons.

*“The estimated location-averaged R_t_ in Thailand usually peaked at the middle of each year with the peak value exceeding 2.00. Sri Lanka had two Rt peaks per year, with peak values lower than those in Thailand. The highest R_t_ in Thailand and Sri Lanka was 3.31 and 3.03 in week 22 of 2014, surpassing those of the other two countries. R_t_ in Singapore reached a peak of 2.15 in week 18 of 2019, while the highest value in Malaysia was observed as 1.79 in week 26 of 2014.” (Track changes: page 12, line 225-231)*

Q7: Figure 1 and caption – the caption could use some additional explanatory information. For example, given that the time-series are not showing years, are these average weekly incidences/temperature/rainfall estimates? Are these showing country-aggregated estimates or are these capturing the range of values across the locations within each country?

A7: The values showed in Fig 1 are observed location-averaged weekly values for the same week of each year across all included locations in the country. We have revised the caption to make it more comprehensive.

*“Each box in the plot represents the location-averaged weekly values for the same week of each year across all included locations in the country. The line within the box denotes the median value, while the whiskers on the top and bottom of each box represent the maximum and minimum location-averaged values observed for that week over the span of nine years.” (Track changes: page 12, line 216-220)*

Q8: Figure 2 and caption: is the dashed line meant to reference Rt =1? Is there any order to the listing of the locations within each country (i.e. by population, incidence, geographic location)? If these are estimated, are they using the observed data from 2012-2020 or are these predictions?

A8: We have revised the plots in this manuscript and ordered the locations within each country by dengue fever incidence (from the highest to the lowest). Figure 2 shows the estimated yearly dengue fever peak reproduction number and epidemic duration. These two variables were calculated from observed data from 2012-2020 by renewal equation. We also revised the caption to clarify the variables showed in this figure.

*“Fig 2. Estimated mean and range of annual peak Rt and epidemic duration. DF epidemic duration was defined as number of weeks with Rt higher than unity in a year. WPKL&Putrajaya: Wilayah Persekutuan Kuala Lumpur & Putrajaya. P.Pinang: Pulau Pinang. Locations within each country are ordered from the highest incidence to the lowest. Observed nine-year peak Rt values are used to calculate the mean and range of annual peak Rt, while the epidemic durations of each of the nine years are used to calculate the mean and range of annual epidemic duration.” (Track changes: page 12, line 233-239)*

Q9: Page 11, line 213-216: First mention of correlation coefficients as part of the model prediction evaluation - Please add these metrics into the Methods. Some additional clarification would help understand the model performance. For example, are these from the testing data, comparing the observed to predicted estimates? Perhaps some mention of which models corresponded to the range of RMSE and correlation values presented would help understand which models performed better.

A9: The estimation of correlation coefficient was added into the Methods section. In this study, we divided the 9-year data into two parts, the first six year was used to build the model, while the remaining three years data was used to evaluate the model’s forecasting performances (i.e. the RMSE and correlation coefficient). We have revised the manuscript and clarified the relative calculations.

*“Weekly dengue fever data from 2012 to 2017 was used to train the model. Data from 2018 to 2020 was then used to validate the prediction performance of the model. A total of 49 models were constructed in each location, and the model with the lowest Root Mean Square Error (RMSE) and correlation coefficients in the model fitting and forecasting was selected.”*

*“Among the 30 models, the RMSEs for fitting and forecasting varied between 0.14 (Selangor, model with temperature up to 6 weeks lag and rainfall up to 6 weeks lag) and 1.01 (Galle, model with temperature up to 4 weeks lag and rainfall up to 6 weeks lag), while the correlation coefficients ranged from 0.10 (Hambantota, model with temperature up to 5 weeks lag and rainfall up to 4 weeks lag) to 0.86 (Chiang Rai, model with temperature up to 5 weeks lag and rainfall up to 6 weeks lag)” (Track changes: page 13, line 242-248)*

Q10: Page 14, lines 266-268: In which ways (both directions and magnitudes) are transmission potential expected to change?

A10: Thanks and we have revised the sentence as following,

*“The peak DF transmission potential was projected to increase by up to 1.80 or decline to up to 1.82 under high emission scenario, and the change would show within-country and between-country variations.” (Track changes: page 15, line 307-309)*

Q11: Page 15: lines 284: Though the focus is on climate, it would be worth adding in text to discuss non-climate factors that could impact transmission intensity and duration. For example, how much might changes in susceptibility or demographic changes in these projected populations also impact future transmission (e.g., <https://www.pnas.org/doi/10.1073/pnas.2115790119>) or comparisons to other papers that have incorporated non-climate aspects into projections (e.g. https://doi.org/10.1038/s41467-023-43954-0)?

A11: We have added the following sentence to indicate other non-climate factors that may affect dengue fever transmission, such as demographic changes. The suggested reference provides strong evidence that the demographic transition would drive disease transmission as well, and it has been appropriately cited.

*“Additionally, changes of other non-climate factors such as demographic and economic situations may also affect the dengue fever transmission projections in the future [34, 35]. ” (Track changes: page 16, line 325-327)*

Q12:Page 15, line 299: A reference for increased severe risk is needed – this recent paper may be useful: <https://www.pnas.org/doi/10.1073/pnas.0709029105>. Some expansion on why there is believed to be a potential shift in risk of severity would help.

A12: We have revised the description to indicate the potential contributors of the higher disease risk in the future, particularly for populations with limited exposure to past dengue outbreaks. The following sentence and reference listed above have been cited:

*“This change pattern partially be attributed to factors including cross-immunity or annual/interannual serotype fluctuation that increase the severity of the disease while the DF transmission declines [36].” (Track changes: page 17, line 342-344)*

**Minor comments:**

Q13: Page 5, line 90: “shorted extinct incubation period” – perhaps meant extrinsic incubation period?

A13: Sorry for the typo, it should be “extrinsic incubation period”. *(Track changes: page 5, line 90)*

Q14: Page 7, line 118-119: are these probable/confirmed cases? Reported symptomatic or hospitalized?

A14: The cases are confirmed by public/private health care institutes and reported to the national health department, which is responsible for releasing number of the cases per week. As this study collected data from national official reports, the cases should be confirmed by the dengue fever diagnose diagnostic criteria, whether the case are hospitalized or have symptoms.

Q15: Page 8, line 139: Do the periods reported reflect the mid-point of the years of each period? Please clarify.

A15: We used the average values to reflect the climate change scenarios within each period. Although we used the mid-point to represent the corresponding time period (e.g., 2030s referred to 2020-2040 average), it was average, but not the mid-point.

Q16: Page 10, line 189-197: Though the averages for incidence/temp/rainfall are reported, providing the IQR values for each country, and perhaps the location with the low and high values would be helpful to better understand how much variability in these there are across the locations.

A16: We have revised the description of the basic characteristics to show more variations.

*“The country-specific nine-year averaged DF incidence was around 25.00 cases per 10,000 people in Singapore, Sri Lanka, and Malaysia, while Thailand had a significantly lower DF incidence rate of 18.21 cases per 10,000 people. Across the 30 included locations, the highest disease incidence (72.35 per 10,000 people) was estimated at Selangor, Malaysia, while the lowest rate (6.56 per 10,000 people) was found at Sarawak, Malaysia. The nine-year average weekly rainfall ranged from 26.86mm (interquartile range, IQR=38.93mm) in Thailand to 47.47mm (IQR=54.07) in Malaysia. The highest and lowest weekly total rainfall were 69.00mm in Ratnapura and 19.90mm in Hambantota, Sri Lanka. The mean temperature was around 27.00°C in these locations, except for Badulla that had an average mean temperature of 23.10°C (see S4 Table for details).” (Track changes: page 11, line 201-211)*

Q17: Figure 2: Adding the peak week as a text to the peak week plot could be helpful since its also referenced in the text.

A17: We have added data labels in Fig 2. Below is the new plot:

*“Fig 2. Estimated mean and range of annual peak Rt and epidemic duration. DF epidemic duration was defined as number of weeks with Rt higher than unity in a year. WPKL&Putrajaya: Wilayah Persekutuan Kuala Lumpur & Putrajaya. P.Pinang: Pulau Pinang. Locations within each country are ordered from the highest incidence to the lowest. Observed nine-year peak Rt values are used to calculate the mean and range of annual peak Rt, while the epidemic durations of each of the nine years are used to calculate the mean and range of annual epidemic duration.” (Track changes: page 12, line 233-239)*
