# Supplementary material for: Projection of dengue fever transmissibility under climate change in South and Southeast Asian countries
Source: PLoS Negl Trop Dis. 2024 Apr 29;18(4):e0012158. doi: 10.1371/journal.pntd.0012158 (PMC11081495; doi:10.1371/journal.pntd.0012158)
Supplement: Attachment — Submitted filename: response to reviewers_clean.docx [file pntd.0012158.s012.docx]

**Response to Reviewers’ Comments**

##Reviewer 2

Q1: The revised version adds good additional detail in the methods and results, and the changes in the Figures help to understand the results better. The authors did a nice job of address my initial comments and I have no further requested changes.

A1: Thank you for reviewing our manuscript. Your comments were incredibly valuable in shaping the final version of the manuscript, and we truly appreciate the time and effort you dedicated to reviewing it.

\## Reviewer 3

The study collected data on weekly dengue fever (DF) incidence, mean daily temperature, and rainfall data at 30 locations in four countries - Malaysia, Singapore, Thailand, and Sri Lanka from 2012 to 2020, and used generalized additive and three Shared Socioeconomic Pathways to estimate the future peak and duration of DF transmission, which is critical for DF prevention and public health wellness. Nevertheless, the manuscript requires minor revision due to the concerns raised below.

Q1: The authors chose 30 places from four nations for the relevant study; can these points from each country reflect all elements of the entire country due to geographical differences?

A1: Thank you for highlighting the potential impact of geographical differences on the representativeness of our chosen locations. We acknowledge that the selected locations may not fully represent entire country, as their selection was determined by their data availability. For instance, the chosen settings in Thailand predominantly fall within the central and northern regions, with the lack of areas in the warm and wet southern regions. Other geographic background such as economy and health care resources may also differ between those selected and not-selected locations in this country. Consequently, the findings from the included locations may not provide a comprehensive depiction of conditions throughout Thailand. Nevertheless, with using multi-settings (rather than a single site in a country), we still believe our results offer valuable insights into the changing trend of DF transmissibility under climate change in the future. We have revised the manuscript by acknowledging this limitation:

*“Another limitation arises from the selection of study locations, which were primarily determined by data availability. The diverse socioeconomic characteristics and other factors present in both the included and excluded locations may restrict the overall representativeness of each country's landscape. Nonetheless, with using multi-settings, the study's findings can offer valuable insights into the changing trends of dengue transmissibility under the influence of climate change” (Track changes: page 20, line 412-417)*

Q2: Climatic circumstances comprise a variety of elements, and the authors are confined in their study of the effect of climate on DF incidence to rainfall and temperature.

A2: Thanks for the comment. In this study, we focused specifically on the change of temperature and rainfall due to their well-documented associations with dengue fever transmission. Compared to other climate factors, the temperature- and rainfall-dependent vector traits, such as egg development rate, biting rate, and intrinsic incubation period, have been extensively studied by various laboratory and field research, and the predicted dengue fever transmission pattern would be easier to be interpreted by these established relationships. On the other hand, temperature and rainfall are two of the significant determinants for dengue fever spread, and effects of other factors may be linked to temperature and rainfall. For instance, previous studies suggest that the impact of humidity on DF infection could vary with changing temperature [[10.1590/S0085-56262010000300021](https://doi.org/10.1590/S0085-56262010000300021)]. Therefore, focusing on these two major meteorological determinants would provide fundamental insights into the potential DF infection under climate change. A discussion has been included in the manuscript:

*“Firstly, only temperature and rainfall were used as indicators of climate change to project DF transmission in the future, leaving other climate variables, such as wind speed, unaccounted for. Despite data availability, the main reasons for this limitation are the well-documented temperature-/ rainfall-DF relationships and the determined impact of these two variables on shaping DF transmission. Additionally, only population growth was considered in projecting future DF transmission potential due to the lack of other socioeconomic variables. We acknowledge that overlooking other influencing factors may reduce the accuracy of our estimation [40]. While the results of this study are promising as we focused primarily on the changes in dengue fever transmission potential throughout this century, a more comprehensive estimation is needed to enhance our understanding of future DF infection risks.” (Track changes: page 19-20, line 397-408)*

Q3: DF is a major public health concern. We should not only manage greenhouse gas emissions, but also implement more effective strategies and measures to eliminate DF, which should be discussed by the authors.

A3: We have included a new paragraph to address the potential implications and proposed interventions for dengue fever prevention and control in the specified region. In summary, it is advised to conduct revised location-specific assessments of DF transmission potential to facilitate the development of customized disease prevention and control measures in a timely manner. Furthermore, the necessity of both national and international regulations to mitigate global warming is emphasized, as this would lead to a decrease in DF risk in the future. Lastly, international collaborations are recommended for controlling DF spread on a global view.

*“Implications and interventions for DF prevention and control in South and Southeast Asia should also be mentioned. Firstly, the spatial heterogeneity of future changes in DF transmission potential highlights the necessity of estimating location-specific dengue infection risks and developing and imposing well-tailored disease prevention and control strategies. These may involve allocating additional healthcare resources to areas with higher DF transmission risk, as well as increasing investment in insecticide spraying and removing stagnant water. Secondly, projections of varying DF spread risks under different climate change scenarios highlight the sensitivity of DF transmission to climate fluctuations. Therefore, it is advisable to strengthen surveillance and monitoring efforts, as well as conduct dynamic assessments of DF infection risk to track the evolving nature of dengue dynamics and facilitate prompt response measures. Thirdly, the predicted warmer and wetter environments in specific regions may favor future DF transmission, emphasizing the significance of limiting global warming to a manageable range. The need for national and international regulations, such as reducing greenhouse gas emissions and promoting the use of clean and sustainable resources to mitigate climate change, is apparent. Lastly, collaborating with international partners to exchange best practices and coordinate efforts in addressing the global challenge of DF would also bring substantial benefits.” (Track changes: page 18-19, line 367-384)*
